# Phase Transitions of Isotropic to Anisotropic Biocompatible Lipid-Based Drug Delivery Systems Overcoming Insoluble Benznidazole Loading

**DOI:** 10.3390/ijms17070981

**Published:** 2016-06-30

**Authors:** Letícia Streck, Víctor H. V. Sarmento, Paula R. L. Machado, Kleber J. S. Farias, Matheus F. Fernandes-Pedrosa, Arnóbio Antônio da Silva-Júnior

**Affiliations:** 1 Laboratory of Pharmaceutical Technology and Biotechnology, Department of Pharmacy, Federal University of Rio Grande do Norte, UFRN, Gal. Gustavo Cordeiro de Farias, Petrópolis, 59.072-570 Natal, RN, Brazil; leticiastreck@gmail.com (L.S.); mpedrosa@ufrnet.br (M.F.F.-P.); 2 Department of Chemistry, Federal University of Sergipe, UFS, Alberto Carvalho Campus, Av. Vereador Olímpio Grande, 49.500-000 Itabaiana, SE, Brazil; vhsarmento@gmail.com; 3 Immunology Laboratory, Department of Clinical Analysis and Toxicology, Federal University of Rio Grande do Norte, UFRN, Gal. Gustavo Cordeiro de Farias, Petrópolis, 59.072-570 Natal, RN, Brazil; paulamachado2@hotmail.com (P.R.L.M.); kfarias3@hotmail.com (K.J.S.F.)

**Keywords:** nanoemulsions, liquid crystals, biocompatible colloidal carriers, rheology, lipid-based drug delivery systems, benznidazole

## Abstract

Previous studies reported low benznidazole (BNZ) loading in conventional emulsions due to the weak interaction of the drug with the most common oils used to produce foods or pharmaceuticals. In this study, we focused on how the type of surfactant, surfactant-to-oil ratio *w*/*w* (SOR) and oil-to-water ratio *w*/*w* (OWR) change the phase behavior of different lipid-based drug delivery systems (LBDDS) produced by emulsion phase inversion. The surfactant mixture composed of soy phosphatidylcholine and sodium oleate (1:7, *w*/*w*, hydrophilic lipophilic balance = 16) stabilized medium chain triglyceride in water. Ten formulations with the clear aspect or less turbid dispersions (five with the SOR ranging from 0.5 to 2.5 and five with the OWR from 0.06 to 0.4) were selected from the phase behavior diagram to assess structural features and drug-loading capacity. The rise in the SOR induced the formation of distinct lipid-based drug delivery systems (nanoemulsions and liquid crystal lamellar type) that were identified using rheological measurements and cross-polarized light microscopy images. Clear dispersions of small and narrow droplet-sized liquid-like nanoemulsions, Newtonian flow-type, were produced at SOR from 0.5 to 1.5 and OWR from 0.12 to 0.4, while clear liquid or gel-like liquid crystals were produced at SOR from 1.5 to 2.5. The BNZ loading was improved according to the composition and type of LBDDS produced, suggesting possible drug location among surfactant layers. The cell viability assays proved the biocompatibility for all of the prepared nanoemulsions at SOR less than 1.5 and liquid crystals at SOR less than 2.5, demonstrating their promising features for the oral or parenteral colloidal delivery systems containing benznidazole for Chagas disease treatment.

## 1. Introduction

Several research areas have considered the manipulation of matter at the nanoscale due to the flexibility and innumerable opportunities that nanotechnology can offer for different applications. Lipid-based drug delivery systems (LBDDS), such as nanoemulsions (NE), have been successfully applied in the food, beverage, chemical, cosmetic and pharmaceutical industries due to their ability to load non-polar ingredients in small-sized oil droplet dispersions [1,2]. Interesting characteristics, such as stability, optical translucency and solvent capacity make these LBDDS a unique nanocarrier for poorly water-soluble drugs [3,4]. In addition, possible loading of specific targeting molecules, such as cholesterol or folic acid, can improve drug distribution in the tissues affected by cancer and reduce side effects [5]. Probably, the use of the same strategy for the treatment of intracellular infections caused by unicellular parasites of the *Trypanosoma* genus can improve the efficacy of antichagasic drugs. Chagas disease is one of the most important public health problems in Latin America, causing morbidity, long-term disability and mortality [6].

Benznidazole (BNZ) (*N*-benzyl-2-nitro-1-imidazole acetamide) is the most common drug for Chagas disease treatment, administered in tablet pharmaceutical dosage form by oral route. However, its poor aqueous solubility and rapid elimination from blood limits oral bioavailability, causing side effects and efficacy problems due to the drug resistance of the parasite [7]. Biocompatible BNZ-loaded NE can solve the absence of an oral liquid pharmaceutical dosage and make possible the use of some strategies for targeting cells infected by the parasite, including the parenteral route. Although BNZ is considered a non-polar drug (partition coefficient of about 0.9), previous studies have revealed that this drug is practically insoluble in both mineral and almond oils [8]. Furthermore, the weak interaction of BNZ with soybean oil-in-water (O/W) emulsions was also reported previously [9].

Hence, the efficient BNZ loading into O/W LBDDS is really a challenge for pharmaceutical nanotechnology. Nevertheless, some important parameters, such as surfactant-to-oil ratio (SOR), oil-to-water ratio (OWR) and even surfactant or oil phase, and the preparation procedure can be optimized in order to solve drug loading limitations, because drug molecules can interact with different hydrophilic, lipophilic or interface domains of the nanoemulsions [10,11].

Among available methods to produce pharmaceutical, cosmetic and food emulsions, the emulsion phase inversion (EPI) method has been successfully applied to obtain small and narrowed-sized nanoemulsions. Thus, important parameters, such as the kind of oil or surfactant, surfactant-to-oil ratio (SOR), presence of surfactant in oil or aqueous phase, titration method and involved energy (low or high energy), should be well standardized [12]. 

Generally, hydrophilic surfactants produce an efficient droplet breakup during phase titration. In addition, a surfactant pair with an ideal hydrophilic-lipophilic balance (HLB) can produce better results for this purpose. Depending on the composition, the aspect of different LBDDS can be monitored by using pseudo-ternary phase diagrams. Colloidal dispersions, such as emulsions, nanoemulsions and microemulsions, present some noticeable differences, which involve thermodynamic stability and physical aspects. The O/W emulsions are thermodynamically unstable, opaque liquid isotropic dispersions of oil droplets in water stabilized by surfactants, which tend to flocculate and coalesce with storage. These flow-resistant and opaque emulsions are routinely classified in the pharmaceutical industry as creams. Microemulsions and nanoemulsions are optically clear or only slightly turbid isotropic dispersions, with small droplet size (nanometer scale, generally a droplet size < 200 nm). Microemulsions are stable thermodynamically, while nanoemulsions are only kinetically stable due to the involved residual free energy.

Small droplet-sized nanoemulsions have demonstrated superior stability during storage due to the reduced effect of gravity over colloidal dispersion. The Brownian motion of particles seems to be sufficient to overcome gravitational separation, avoiding flocculation and coalescence [12,13]. However, a large surfactant-to-oil ratio can be required to obtain these systems, which can lead to the formation of liquid crystals (LC), which are sometimes clear, but essentially anisotropic systems (lamellar or hexagonal phases) or with high viscosity (cubic phase). Sometimes, nanoemulsions are incorrectly classified in the pseudo-ternary phase diagrams. This makes it necessary to investigate and clarify these isotropic-to-anisotropic phase transitions. Generally, relevant alterations in the flow behavior, viscosity and microstructure of the LBDDS involve any isotropic-to-anisotropic phase transition that changes the drug release rate and the suitable administration route. The physicochemical properties of the compounds and the surfactant-to-oil ratio are the main factors that determine the microstructure of the colloidal dispersion and, therefore, the rheological properties of the nanoemulsions [14,15,16].

In this study, medium chain triglycerides (MCT) were associated with a surfactant pair composed of soy phosphatidylcholine (SPC) and sodium oleate (SO) with the best HLB to produce oil-in-water BNZ-loaded biocompatible nanoemulsions. The use of MCT as the oil phase was due to their common use in parenteral emulsions with assured safety. The pseudo-ternary phase diagram permitted assessing the effects of the surfactant-to-oil ratio and oil-to-water ratio on the phase behavior and structural properties of different LBDDS. The phase transitions were visually assessed and well characterized by rheological measurements, cross-polarized light microscopy (CPLM) and dynamic light scattering (DLS). The *in vitro* Vero cell viability model evaluated the biocompatibility of different LBDDS. 

## 2. Results

**Figure 1:**
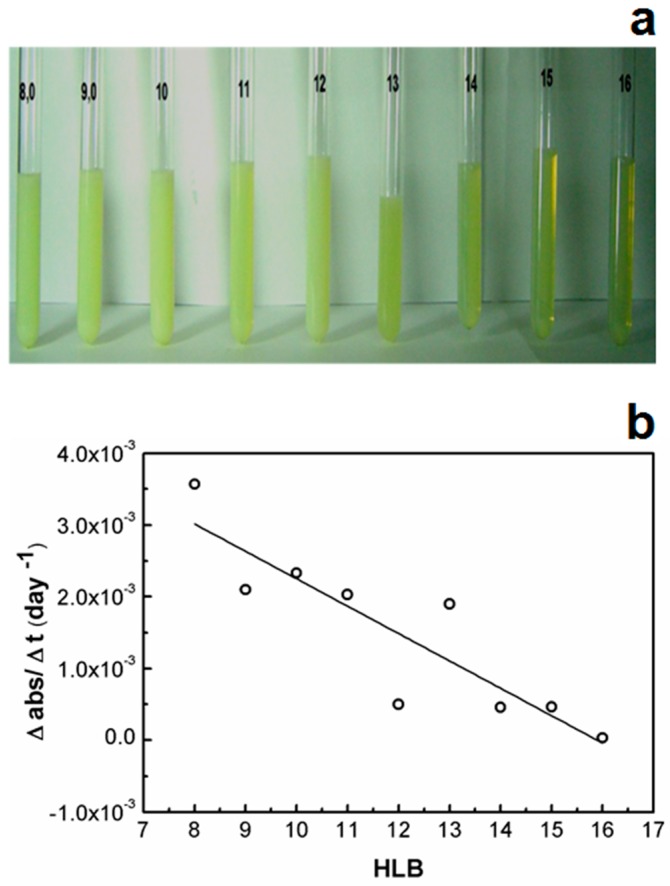
(**a**) Transparency at 24 h and (**b**) turbidity variation during 30 days as a function of the SPC/SO ratio at different HLB. Note: the samples contain MCT (10% *w*/*w*), SPC/SO (10% *w*/*w*) and purified water (80% *w*/*w*).

The ability of different SPC/SO mixtures (HLB = 8 to 16) to produce clear or slightly turbid colloidal dispersions, containing the oil phase at 10% *w*/*w* in water, was assessed, as shown in Figure 1a. The HLB values between 12 and 16 produced the best results. In the specific studied EPI method, the water was titrated in a mixture containing the oil phase with surfactants. Consequently, the higher affinity of the surfactant by the aqueous phase enables it to efficiently stabilize O/W emulsions. 

The samples at different HLB values presented pH ≈ 10.3 during the whole experiment period (after the 1st, 7th and 30th days). In this study, the formulations were not buffered in order to avoid the enhancement of the ionic strength, which could perturb the phase behavior in a composition-dependent manner. For further *in vivo* applications, the optimized formulations should be buffered to a more physiologically-relevant pH according to the desired administration route, with the physicochemical properties monitored and compared to the data presented in this approach. In this initial screening, the short time (30 days) and storage temperature (25 °C) did not induce relevant pH changes. In addition, another investigated parameter was the refraction index (RI). The RI remained about 1.35. The relative turbidity increment (Δabs/Δt) was determined by the ratio of the difference of absorbance measured at the 30th day and the first day by the time of study (30 days) (Figure 1b).

### 2.1. Effect of SOR and OWR on the Aspect and the Rheological and Optical Properties of LBDDS

**Figure 2:**
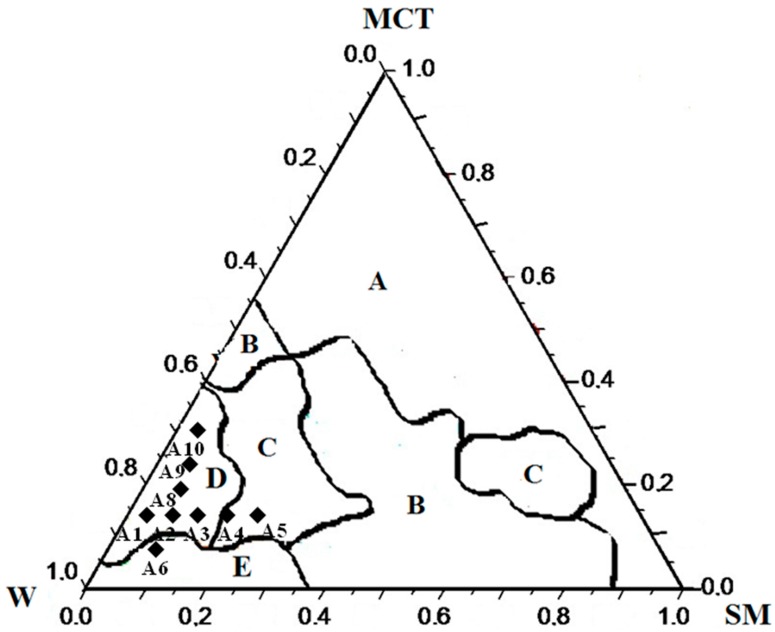
Pseudo-ternary phase diagram involving MCT stabilized with the surfactant mixture (SM) SPC/SO at HLB = 16 in purified water (W): (A) phase separation, (B) creams, (C) liquid crystals, (D) nanoemulsions and (E) emulsions.

The effect of the surfactant-to-oil ratio (SOR) or oil-to-water ratio (OWR) on the aspect and possible phase transitions among different lipid-based drug delivery systems made their classification possible in the pseudo-ternary phase diagrams according to the light dispersion aspect and flowability (Figure 2). The phase separation (A) was observed mainly at a small water proportion in the mixtures. The opaque and viscous flow-resistant emulsions were classified as O/W or W/O creams (B), while clear ones were classified as liquid crystals (C). The clear and less turbid liquid dispersions were classified as O/W or W/O nanoemulsions (D); and finally, the opaque liquid dispersions were classified as O/W or W/O emulsions. 

**Figure 3:**
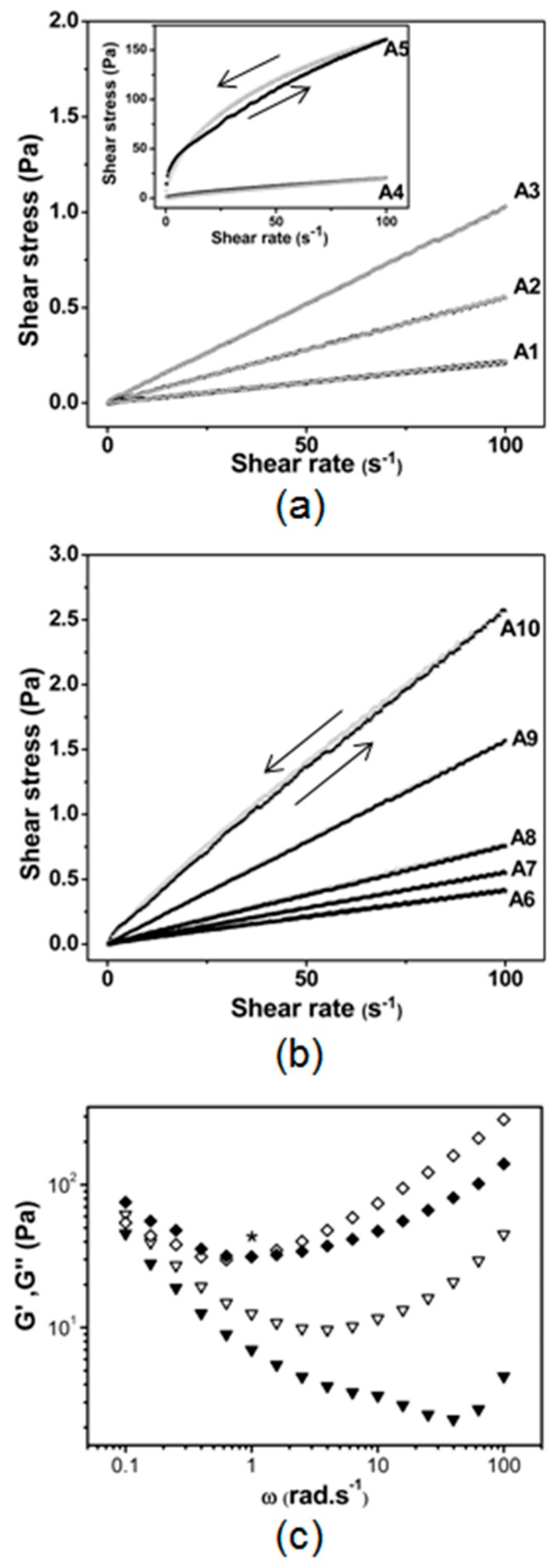
Effect of (**a**) SOR (A1 to A5) and (**b**) OWR (A6 to A10) on flow behavior; (**c**) viscoelastic behavior of non-Newtonian samples A4 (▼) and A5 (♦). Storage moduli (full symbol) and loss moduli (empty symbol) as a function of angular frequency. Note: the symbol * indicates the crossover.

Five formulations were selected in the pseudo-ternary phase diagram for evaluating the effect of surfactant-to-oil ratio (SOR = 0.5 to 2.5) (A1, A2, A3, A4 and A5) and five for the oil to water ratio (OWR = 0.06 to 0.4) (A6, A7, A8, A9 and A10) on the structural features and the ability to load the insoluble drug BNZ. The formulations A2 and A7 had the same composition. The effect of SOR and OWR on the rheological behavior (flow curves) for these free drug-loaded lipid-based drug delivery systems is shown in Figure 3a,b. The ascending curves (full symbol) recorded at a shear rate ranging from 0.25 to 100 s^−1^ and 100 to 0.25 s^−1^ for the descending curves (empty symbol) made it possible to observe any time-dependent change in viscosity for the sample A5. Additionally, the flow parameters for both free drug and drug-loaded lipid-based drug delivery systems were also assessed according to *τ* = *K* ($\dot{\gamma }$) *^n^*, in which τ is the shear stress, $\dot{\gamma }$ is shear rate, *K* is the consistency index and *n* is the flow index. Experimental data fitted using the power law model (*R*^2^ > 0.99) are shown in Table 1. Despite the transparent aspect of A1 to A5 samples, the SOR rising changed the flow behavior of samples (Figure 3a). The samples A1 to A4 were liquid-like dispersions, but the samples A4 and A5 exhibited flow index *n* < 1.0, with considerable enhancement of the consistency index *k*, characteristic of pseudoplastic systems, such as liquid crystals. SOR values smaller than 1.5 (samples A1 to A3) exhibited the Newtonian flow type (*n* ≈ 1), which is a signature of nanoemulsions. The rise of the oil-to-water ratio (OWR) from 0.06 to 0.40 for samples A6 to A10 did not induce any change in the flow behavior (Figure 3b and Table 1). 

The oscillatory tests carried out for samples A4 and A5 confirmed the non-Newtonian flow and revealed the influence of SOR on the viscoelastic properties of the samples (Figure 3c). The representative plots of storage (G′) and loss (G″) moduli *versus* angular frequency (ω) demonstrated that the A4 sample exhibited liquid-like behavior (G′ < G″) for the entire ω. The crossover (G′ = G″) at ω = 1 rad·s^−1^ indicated a gel-like behavior (G′ > G″) at low frequencies (ω < 1 rad·s^−1^) for the sample A5, with liquid-like behavior (G′ < G″) only at high frequencies (ω > 1 rad·s^−1^).

**Figure 4:**
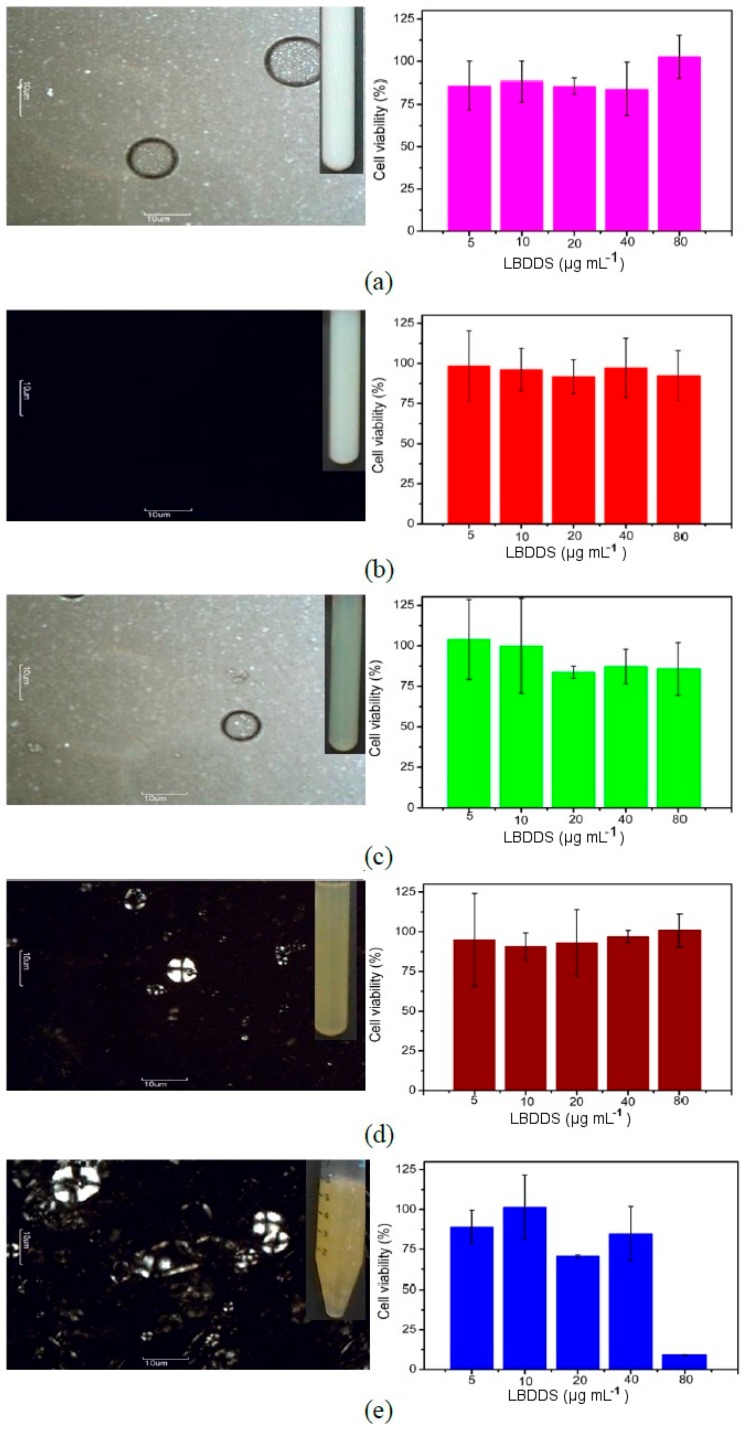
Isotropic/anisotropic behavior assessed by CPLM (**left**, magnification 400×) and the respective cell viability assays for the different LBDDS (SOR of (**a**) 0.5, (**b**) 1.0, (**c**) 1.5, (**d**) 2.0 and (**e**) 2.5) against Vero cells after 12 h at 37 °C (**right**). Note: data of the relative cell viability are expressed as the mean ± SD (*n* = 3).

**Figure 5:**
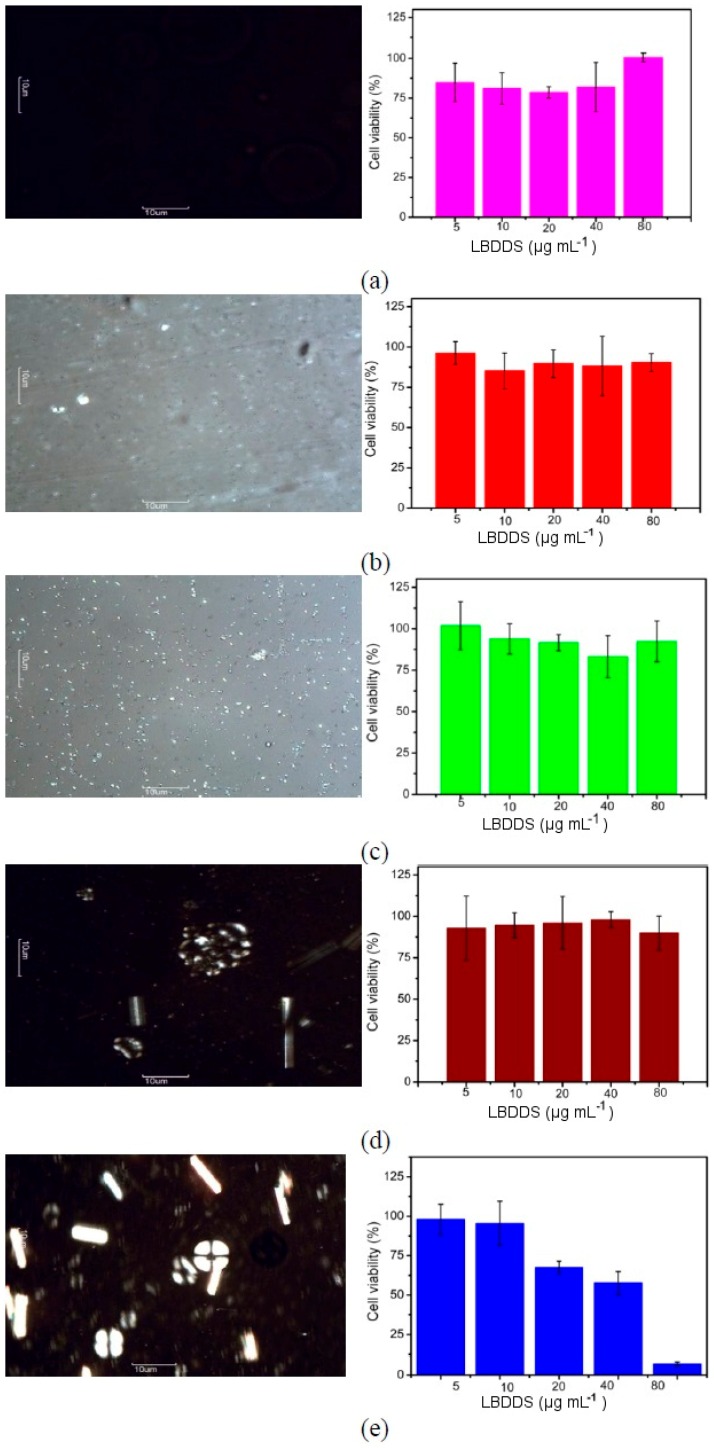
Isotropic/anisotropic behavior assessed by CPLM (**left**, magnification 400×) and respective cell viability assays for the different BNZ-loaded LBDDS (SOR of (**a**) 0.5, (**b**) 1.0, (**c**) 1.5, (**d**) 2.0 and (**e**) 2.5) against Vero cells after 12 h at 37 °C (**right**). Note: data of the relative cell viability are expressed as the mean ± SD (*n* = 3).

These important differences among the free drug-loaded nanoemulsions (A1 to A3) and liquid crystals (A4 to A5) were clarified using the cross-polarized light-microscopy images (Figure 4). The LBDDS with the surfactant-to-oil ratio ranging from 0.5 to 1.5 (A1–A3) exhibited the characteristic dark field (Figure 4a–c), confirming the specific isotropic behavior, a signature of nanoemulsions. The samples prepared with SOR of about 2.0 to 2.5 (A4 to A5) exhibited structures similar to Maltese crosses (Figure 4d,e), a feature of the liquid crystal lamellar type. The drug loading in these systems did not change the behavior identified in both nanoemulsions (Figure 5a–c) and liquid crystals (Figure 5d,e). In addition, all investigated OWR showed isotropic behavior (data not shown), characteristic of emulsion (A6) and nanoemulsions (A7 to A10).

The rheology and CPLM images exposed the clear distinction between nanoemulsions and liquid crystals in the pseudo-ternary phase diagram, permitting a correct interpretation of the physicochemical parameters, such as diameter, polydispersity index (PdI) and zeta potential for the nanoemulsions (Table 1). The liquid crystals (samples A4 and A5) do not exhibit droplets or particles in their structure, but the surfactants are arranged as lamellar phases in colloidal dispersions with considerable consistency index. Thus, the DLS and zeta potential measurements were not carried out for these samples. Their dilution could induce their phase transition to nanoemulsions or liposomes.

DLS results confirmed that the selected surfactants for the EPI method produced nanoemulsions with a small and narrow droplet size distribution. All samples exhibited PdI values lower than 0.3.

### 2.2. Effect of SOR and OWR on Drug Loading

**Figure 6:**
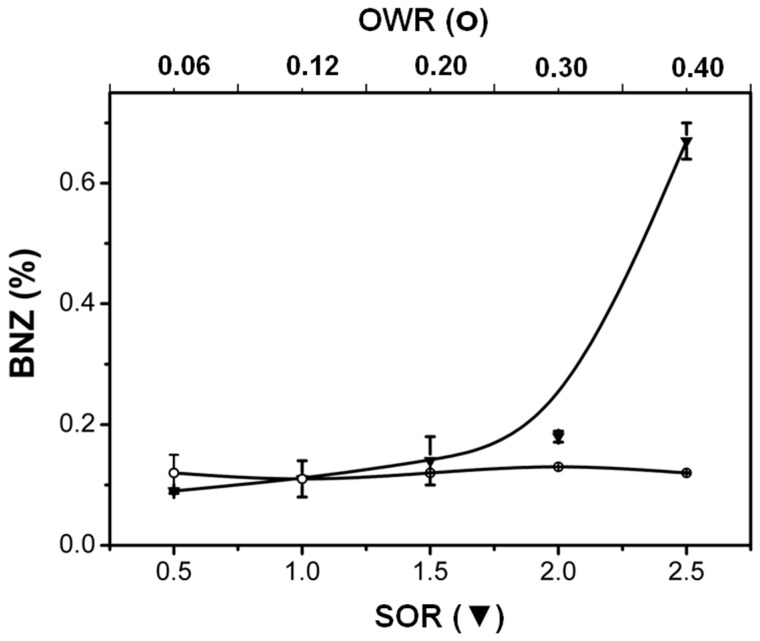
BNZ loading as a function of the surfactant-to-oil ratio (SOR) or oil-to-water ratio (OWR). Note: data are expressed as the mean ± SD (*n* = 3).

After the BNZ loading, the samples prepared at distinct SOR (A1, A2 and A3) had their diameter increased, compared to free drug loaded samples (Table 1). For the samples A6, A8 and A10, the droplet size decreased with drug loading, suggesting that a considerable part of the drug partitioned to the oil-to-water interface, resulting in more compact films. Furthermore, the effect of the composition on drug loading for the different lipid-based drug delivery systems is shown in Figure 6. Despite the non-polar character of BNZ, the enhancement of the oil-to-water ratio did not increase drug loading, proving its weak interaction with the MCT oil phase. This information was also supported by rheological measurements (Table 1), which demonstrated that BNZ-loaded LBDDS exhibited the same Newtonian flow behavior (*n* ≈ 1) and similar consistency index *K* compared to the respective free drug-loaded LBDDS. 

### 2.3. Cytotoxicity Assays

The enhancement of the SOR in the LBDDS could potentially induce cytotoxicity. This study was performed in Vero cells by the MTT assay for the different free drug-loaded nanoemulsions and liquid crystals (Figure 4) and the same drug-loaded formulations (Figure 5) at distinct concentrations (5, 10, 20, 40 and 80 μg·mL^−1^). The MTT assay did not reveal cytotoxicity dependent on the type of structure of LBDDS, but on surfactant concentration. All free drug-loaded nanoemulsions prepared at surfactant-to-oil ratios ranging from 0.5 to 1.5 (A1 to A3) (Figure 4a–c), and the liquid crystal produced at SOR = 2.0 (A4) (Figure 4d) did not show cytotoxicity for all of the tested concentrations (5 to 80 μg·mL^−1^). However, the free drug-loaded liquid crystal prepared at the highest SOR (A5) showed cytotoxicity for the highest tested concentration (80 μg·mL^−1^) (Figure 4e). The experiment performed with drug-loaded formulations revealed the same behavior (Figure 5). In addition, nanoemulsions formulated at different oil-to-water ratios (0.06 to 0.4) did not show any cytotoxic effect (data not shown). 

## 3. Discussion

The main differences among microemulsions, nanoemulsions and emulsions are the physical stability and optical properties. When the transparency is a characteristic of these drug delivery systems, the droplet size is certainly the main relevant parameter to produce clear or slightly turbid colloidal dispersions. Generally, a large amount of surfactant is required to produce transparent nanoemulsions. However, suitable surfactants produce stable and less turbid colloidal dispersions. The best composition of the surfactant mixture (SM) composed of phosphatidylcholine and SO able to induce the formation of clear colloidal dispersions was assessed in this study. The optical clarity of the dispersions increased according to the amount of SO (enhancement of the HLB) (Figure 1a). Hydrophilic surfactant induces the more efficient oil droplet breakup during the emulsion phase inversion, and its association with lipophilic surfactants is applied for the maximum effect in the reduction of the particle size [12,17]. Thus, an ideal mixture of surfactants can easily be tuned by using the HLB value, contributing to produce films of surfactants with sufficient viscosity to avoid coalescence [18,19].

The linear fitting in Figure 2b suggested the high stability for those dispersions prepared with surfactant mixtures with HLB greater than 11, supporting the visual aspect observed at the first 24 h. The turbidity of colloidal dispersions generally increases due to the concentration of droplets, the coalescence, and thereby to the droplet size enhancement. Thus, the surfactant mixture with HLB = 16 was selected to build the pseudo-ternary phase diagram in order to observe the region of clear and transparent colloidal dispersions. 

Figure 2 shows that a considerable region of cream (Region B) occurred between two regions of liquid crystal (Region C). These phase transitions transpired according to the amount of surfactant increased in the LBDDS. This phenomenon revealed the ability of these specific surfactants to form liquid crystals, mainly at high concentrations. Previous studies have demonstrated this property for the sodium oleate [20]. The transitions of liquid crystals to nanoemulsions (Region D) occurred when the surfactant ratio decreased in the mixture. A similar approach was used by Pund *et al.* [21] to obtain resveratrol-loaded nanoemulsions, in which a large NE area in the pseudo-ternary phase diagram was reached by using a surfactant pair composed of Transcutol^®^ and Labrasol^®^. The experimental data showed that the surfactant mixture with suitable HLB was decisive to stabilize the MCT dispersions. The SOR values ranging from 0.5 to 1.5 (A1 to A3) produced liquid-like transparent nanoemulsions, while those greater than 2.0 (A4 and A5) considerably increased the viscosity due to the formation of the structures arranged as liquid crystals.

Despite the sample A4 (SOR 2.0) presenting the transparent liquid-like dispersion in the limit of the nanoemulsion region, the flow index *n <* 1 suggested the possible phase transition of nanoemulsion to liquid crystal, with the simultaneous presence of these two types of structures in the sample. The sample A5 (flow index *n ≈* 0.5) confirmed the behavior. In addition, the sample A5 exhibited anti-thixotropic behavior due to the more ordered arrangement of the highest surfactant ratio after the shearing process. This phase transition directly affects the use and performance of the LBDDS, once that nanoemulsions exhibit the Newtonian flow behavior, because the oil is structured as droplets into the aqueous phase stabilized by surfactants. These characteristics permit their use as drug targeting systems applied by several routes, with the drug-loading and drug release rate generally controlled by droplet size, SOR and OWR [11,12,13,14,15]. On the other hand, the liquid crystals show the non-Newtonian flow behavior with viscoelastic properties, because the oil is loaded among the surfactants’ chains specifically structured as lamellar, hexagonal or cubic phase [16]. In the liquid crystals, the drug loading and drug release rate are controlled by the kind of formed structure (lamellar, hexagonal or cubic phase), and they are generally suitable for topical, transdermal and oral use. Similar behavior dependent on the surfactant ratio was observed in previous studies carried out by Pestana *et al.* [22]. The rheological properties of the biocompatible microemulsions containing the surfactant pair composed of soy phosphatidylcholine:Tween 20 (1:1, *w*/*w*), Captex™ 200 as the oil phase and phosphate-buffered saline as the aqueous phase were studied for the incorporation of amphotericin B, an antifungal drug.

Recently, the formation of anthranilic acid-based wormlike micelles induced by the pH was assessed using rheological measurements. The oscillatory tests proved the transition of the Newtonian dispersions to the viscoelastic dispersions [23]. Figure 3c provided comprehensive information about the viscoelastic behavior of the samples A4 and A5, a signature of the liquid crystals, whereas nanoemulsions had Newtonian behavior. The data of the oscillatory tests suggested two important aspects: first, the sample A4 is in the transition region of the phase diagram, and this viscoelastic behavior can be associated with the formation of the liquid crystals, while the SOR rising led to structured systems with viscoelastic properties. This structural feature affects the feasibility of the LBDDS, such as the drug release rate, stability and possible administration route [24]. This same behavior did not occur when the OWR was enhanced in the emulsion (A6) to nanoemulsions (A7 to A10) (Table 1). Even the samples with OWR of about 0.4 remained as liquid-like dispersions and exhibited the Newtonian flow type (*n* ≈ 1), with *K* < 0.05. This fact demonstrated the desired stabilizer effect of the surfactant pair used. The slight enhancement in the consistency index (*K*) was due to the rise in the droplet number, with a more effective hydrodynamic interaction with the internal phase [25,26].

The CPLM images (Figure 4 and Figure 5) have confirmed the presence of the liquid crystals of the lamellar type in the samples A4 and A5 with or without BNZ loaded. Liquid crystals, such as hexagonal or lamellar phases, have different light-scattering behavior of the nanoemulsions. These dispersions are anisotropic systems due to the well-defined structures formed by surfactants in aqueous medium with the oil phase arranged according to the lipophilic domain. On the contrary, O/W nanoemulsions are isotropic systems, structured by surfactants randomly arranged around an oil droplet [27]. Previous studies described the effect of SOR and internal phase volume on drug loading in order to evaluate the possible domain of the drug arrangement into oil droplets or around surfactant film. However, these studies have not justified the isotropic behavior, inducing misinterpretations of DLS data [28]. 

The DLS data (Table 1) have demonstrated the expected effect of the SOR on the droplet size of the nanoemulsions; both free drug-loaded and drug-loaded nanoemulsions have the mean diameter decreased according to the enhancement of SOR in the samples. However, the zeta potential remained constant, suggesting the possible surfactant saturation around the oil droplets. This surfactant arrangement can lead to oil penetration among surfactant molecules in the alkyl chain region for a minimum and stable droplet size [29,30,31]. During water titration, phase transitions occur involving successive conversion of the W/O nanoemulsion to the bicontinuous phase and finally to the O/W nanoemulsion, demonstrating the importance of the surfactant mixture with suitable HLB to reduce interfacial tension [32,33]. The type and concentration of the surfactants affect the transport rate of oil droplets to the aqueous phase [34]. In the present approach, the composition of surfactant mixture composed of anionic surfactants (HLB) affected their ability to induce the formation of clear dispersions of MCT in water, while their concentration induced the transitions of nanoemulsions (SOR less than 1.5) to liquid crystals (SOR greater than 2.0), showing promising features for BNZ loading.

The effect of BNZ loading in the structural features of the LBDDS was also analyzed. The slight increase of droplet size possibly occurs due the drug dissolution into the internal phase or drug anchoring in the surfactant film around oil droplets [28,35,36]. Previous studies with antitumoral doxorubicin-loaded microemulsions have demonstrated the favorable partition of the hydrophobic non-ionized drug into the oil phase, as an additional fraction of the oil phase added to the microemulsions. A small fraction of the ionized drug partitioned for the surfactant monolayer at the oil-water interface due to the amphiphilic character leading to the droplet size increase [37,38]. The same effect was observed for the antifungal amphotericin B-loaded biocompatible microemulsions [23]. Although BNZ is considered a non-polar molecule (partition coefficient ≈ 0.9), the hypothesis of the drug dissolution into the oil phase was discharged because the same effect did not occur in the experiment with distinct OWR (samples A6–A10). 

The negative zeta potential increased with BNZ loading, assuring the electrostatic stabilization, avoiding the possible flocculation. The drug anchoring in the interface around oil droplets perturbs the arrangement of the monolayer or multilayer of surfactants, stabilizing the oil dispersion [39]. The most effective molecular packing of the emulsifiers at the interface contributes to the decrease in droplet size of the nanoemulsions [40]. In addition, Figure 6 showed the weak interaction of BNZ with the oil phase (samples A6–A10). The BNZ loading increased according to the SOR rising, proving that BNZ interacts with the surfactant film around the oil, in the case of nanoemulsions, a common behavior for insoluble molecules [41]. The rheological measurements also supported this fact (Table 1).

The flow behavior of the BNZ-loaded LBDDS was not altered compared to free drug-loaded LBDDS prepared at a SOR of 0.5 to 2.5. However, the drug loading decreased the consistency index *K* mainly for those samples with a larger amount of BNZ loaded into LBDDS (SOR at 2 and 2.5), confirming that BNZ perturbs the surfactant film. Moreover, the highest investigated SOR (2.5) considerably increased the drug loading (0.67%, *w*/*v*), 34-fold that of the BNZ solubility in water, which demonstrates the superior ability of the liquid crystal lamellar phase-type in solubilizing drugs that interact with surfactant layers. The maximum amount of surfactant (SOR = 1.5) enables the production of a liquid-like nanoemulsion, leading to drug loading of about 0.14% w/v, which was sevenfold that identified in water (0.02%, *w*/*v*). This solubilizing capacity of BNZ was superior to that identified for the ternary complexes with β-cyclodextrin [42] and opaque liquid-like emulsions [9] previously investigated for this purpose.

The *in vitro* cytotoxicity studies in this approach allowed to predict possible effects in further studies with Vero cells infected by the parasites for the design of experiments with animals, and consequently humans. Both free drug-loaded and drug-loaded LBDDS were shown to be biocompatible nanocarriers for BNZ release, specifically the nanoemulsions (oral and parenteral routes) (SOR smaller than 1.5) and liquid crystals (oral route) (SOR higher than 2.0). In addition, both free drug-loaded and drug-loaded liquid crystal formulated at SOR = 2.5 showed a cytotoxicity dependent on dilution. Different types of cells have been used for this purpose. Han and Zhou [43] demonstrated that Fe_3_O_4_ nanoparticles embedded in liposomes reduced the toxicity of the magnetic particles evaluated in HeLa cells at 24 h. Considering a similar assay in Caco-2/TC7 cell line at 4 and 24 h of incubation, Benzaria *et al.* [44] demonstrated the biocompatibility of the O/W submicron emulsions of sesame oil stabilized with polysorbate 80. Chen *et al.* [45] used a surfactant mixture containing SO to stabilize solid lipid nanoparticles that showed the biocompatibility in a HeLa cell model. However, it is not easy to produce biocompatible lipid-based drug delivery systems with a considerable amount of surfactant. Previous studies with W/O/W nanoemulsions prepared with lauroglycol-90 as the oil phase and a surfactant mixture containing polyethoxylated castor oil (Cremophor EL^®^) demonstrated cell viability of about 70% for the highest tested surfactant concentration (about 40% *w*/*w*) in the HT-29 colon cancer line [46]. 

Before using a specific administration route, it is fundamental to predict the interaction of a specific lipid-based drug delivery system with tissues and possible structural changes. The experimental data in this study permitted us to postulate that the optimized formulations are promising for the *in vitro* efficacy studies against the parasite, using the infected Vero cell model. This approach can make it possible to select the best candidates for the studies with animals and humans [47,48,49]. Regarding the possible administration routes, the passage of nanoemulsions by different gastrointestinal tract compartments, or dilution in the plasmatic circulation, for example, can change the droplet size and zeta potential, destabilizing the colloidal dispersion due to the changes in water amount, pH or composition of the medium [50]. 

## 4. Materials and Methods

### 4.1. Materials

MCT (Miglyol^®^ 810, Sasol, Hamburg, Germany) was used as the oil phase. A surfactant mixture (SM) composed of SPC (Epikuron^®^ 200, Cargill, Frankfurt, Germany) and SO (Sigma–Aldrich, St. Louis, MO, USA) was used to stabilize the LBDDS. BNZ (Roche, São Paulo, Brazil) has been provided by the Pharmaceutical Laboratory of Pernambuco-Brazil (LAFEPE, Recife, Brazil). The purified water (0.15 μS·cm^−1^) was prepared by using reverse osmosis purification equipment; Model OS50 LX, Gehaka (São Paulo, Brazil).

### 4.2. Pseudo-Ternary Phase Diagram

Preliminary experiments carried out with formulations containing MCT as the oil phase, the surfactant mixture and purified water at a fixed ratio (10:10:80, *w*/*w*/*w*), allowed to identify the best HLB able to produce clear and less turbid O/W nanoemulsions. The range of HLB investigated for SM containing SPC (HLB = 4.0) and SO (HLB = 18) was calculated by using Equation (1), in which the values of “*x*” and “*y*” ranged from 0 to 1 (*x* + *y* = 1) and corresponded to the surfactant ratio in the surfactant mixture. Measurements of pH, turbidity and refraction index carried out for the different formulations at specific intervals of 72 h during 30 days permitted assessing the stability. Samples were stored at 25 °C during the experiments, and the assays were carried out in triplicate.

HLB*_SM_* = *x* HLB_SPC_ + *y* HLB_SO_ (1)

Once the best HLB was established, suitable SOR and OWR were used in the range of 1:9 (*w*/*w*) to 9:1 (*w*/*w*), respectively. Purified water was slowly titrated with a precise automatic micropipette into the mixtures containing the oil phase and surfactant mixture at controlled temperature (70 ± 2 °C). The titrations were carried out at intervals of 1 min by using an ultrasonic liquid processor (XL 2020 model, Misonix Incorporated, USA) equipped with a titanium alloy horn (3 mm) with a 12.7 × 3.8 cm driving force in an ultrasonic horn of a driving frequency of 20 kHz. The transitions were sharp, reproducible and plotted in delimited points at specific regions in the phase diagram, which were classified according to the visual appearance against a dark background. Liquid-like clear and less-turbid dispersions were classified as O/W or W/O nanoemulsions and opaque liquid-like as O/W or W/O emulsions. Flow-resistant viscous emulsions were classified as O/W or W/O creams; and finally, flow-resistant, clear, viscous and less turbid dispersions were classified as liquid crystals. The samples were stored at controlled room temperature (25 °C) for 24 h before the experimental studies.

### 4.3. Preparation of the Lipid-Based Drug Delivery Systems

Some formulations identified in the pseudo-ternary phase diagrams were used to assess the effect of the composition on the structure and drug loading into these different LBDDS. Firstly, the oil phase was fixed at 10% *w*/*w* and mixed with distinct surfactant mixture (SPC/SO, 1:7, *w*/*w*) amounts, allowing identification of LBDDS at distinct surfactant-to-oil ratios (*w*/*w*): 0.5, 1.0, 1.5, 2.0 and 2.5; namely, as A1, A2, A3, A4 and A5, respectively. In the second experiment, the same surfactant mixture fixed at 10% *w*/*w* was mixed with different oil phase amounts, enabling the identification of LBDDS at distinct oil-to-water ratio (*w*/*w*): 0.06, 0.12, 0.20, 0.30 and 0.40 (A6, A7, A8, A9 and A10, respectively). The same composition was used for the samples A2 and A7. The formulations were prepared in triplicate by using the same conditions for the pseudo-ternary phase diagram. The samples were stirred for 10 min, centrifuged at 1000× *g* for 15 min (Excelsa^®^II centrifuge, 206-BL model, Fanem, São Paulo, Brazil) and stored at controlled room temperature (25 °C) for 24 h before the measurements. 

### 4.4. Drug Loading into LBDDS

The oil phase containing an excess of BNZ was emulsified as described in the preparation of LBDDS. Different samples remained in a thermostated bath at 25 °C, and were shaken in a vortex for 1 min followed by 15 min in an ultrasonic bath, every 12 h during 72 h. After centrifugation (1000× *g* for 15 min), the samples were filtered through an acetate membrane (0.45 μm), and the drug amount was analytically determined by UV-vis spectrophotometry at 315 nm, according to the methodology validated previously [51].

### 4.5. Rheology 

Rheological measurements for the different lipid-based drug delivery systems were carried out using an MCR Rheometer (Anton Paar GmbH, Ostfildern-Scharnhausen, Germany), equipped with a titanium cone and plate geometry (1° angle, 5 cm diameter and a gap of 102 μm between cone and plate). The viscosity of the samples was determined by linear steady-state flow measurements in increasing the shear rate, from 0.25 to 100 s^−1^, for the ascending curve, and 100 to 0.25 s^−1^, for the descending curve, for 5 min at 25 °C controlled by using a Peltier cell. All samples were prepared 24 h before the measurements. The data of the shear cycle were fitted by the power-law model, using the Rheoplus/32 V3.60 software.

The viscoelastic behavior of the samples with the pseudoplastic flow-type was assessed by oscillatory tests carried out by using a frequency sweep from 0.1 to 100 rad·s^−1^ at a constant strain amplitude (1 Pa) at 25 °C. The storage modulus (G′) and loss modulus (G″) were then determined, and the frequency range of the G′ and G″ values was plotted on a logarithmic scale and used to analyze the viscoelastic rheological behavior of the samples. The linear viscoelastic region (the region where the moduli are constant and preserve the structure) was determined using a strain sweep test at 1 Hz.

### 4.6. Cross-Polarized Light Microscopy

Isotropic or anisotropic samples were placed on a glass slide and covered with a coverslip. They were examined under CPLM with 400× magnification in a Jenamed 2 (Carl Zeiss Jena, UK) equipped with a digital camera (Sony, Japan).

### 4.7. Droplet Size and Zeta Potential Measurements 

The mean droplet diameter was calculated by using the cumulative method of analysis, according to the intensity of the light scattered in a particle size analyzer (Brookhaven Instruments, New York, NY, USA), at a 659 nm wavelength, 90° detection angle and at 25 °C. The correlation worked in parallel mode, and the data were analyzed by using Zeta Plus^®^ Particle Sizing Version 3.95 software. Zeta potential (ZP) measurements were performed with the same equipment applying a field strength of about 5.9 V·cm^−1^. Five runs for each sample determined the ZP value with PALS Zeta Potential Analyzer software by using the electrophoretic mobility according to the Helmholtz–Smoluchowski equation. The samples were diluted at 1:100 (*v*/*v*) with purified water.

### 4.8. Cytotoxicity Assay

Vero E6 cells cultured in a Leibovitz (L-15) medium, supplemented with 10% fetal bovine serum, 10% tryptose, 1% streptomycin-amphotericin B at 37 °C under fully-humidified conditions were seeded at a density of 2 × 10^5^ per well in 100 μL of culture medium into a 96-well plate and incubated overnight. After the formation of the monolayer, the culture medium was removed with phosphate-buffered saline (PBS) solution. Different lipid-based drug delivery systems prepared with a surfactant-to-oil ratio at 0.5 to 2.5 *w*/*w* were incubated at 5, 10, 20, 40 and 80 μg·mL^−1^ on the plate for 12 h at 37 °C in triplicate for each dilution using two plates. The medium (200 μL) was aspirated, and the plate was incubated with 50 μL of 3-[4,5-dimethylthiazol-2-yl]-2,5-diphenyltetrazolium bromide (MTT) solution at 1 mg·mL^−1^ for 4 h at 37 °C. After dissolution of the formed formazan crystals by using 100 μL of dimethyl sulfoxide, the absorbance of the plate was measured at 540 nm using a microplate reader (Biotek, Epoch model, USA). The measurements from the untreated cells were used as the control assay. Cytotoxicity, scored as relative cell viability, was defined according to Equation (2).

(2) $$\text{Relative cell viability }\left(\%\right)=\;\frac{\text{optical density of treated cells at }540\text{ nm}}{\text{optical density of untreated cells at }540\text{ nm}}\;\times 100$$

### 4.9. Statistical Analysis

Mean values identified in the experiments were compared by one-way analysis of variance (ANOVA) followed by Tukey´s test for multiple comparisons or Dunnett’s for multiple comparisons *versus* a control group, as well as Student’s *t*-test for pairwise comparisons. A *p* value < 0.05 was required for significance.

## 5. Conclusions

In this study, the poorly water-soluble BNZ drug loading was improved according to the composition and type of lipid-based drug delivery system produced by the EPI method. Clear colloidal dispersions were successfully produced using a suitable surfactant mixture, composed of SPC and SO (1:7, *w*/*w*, HLB = 16). The rise in the surfactant-to-oil ratio (0.5 to 2.5) induced the formation of distinct lipid-based drug delivery systems (nanoemulsions and the liquid crystal lamellar-type) that were identified using rheological measurements and CPLM images. Small and narrow droplet-sized nanoemulsions were prepared even for the highest oil-to-water ratio (0.4). Cell viability studies demonstrated the biocompatibility for all of the prepared nanoemulsions at an SOR of less than 1.5 and liquid crystals at an SOR smaller than 2.5, demonstrating the promising features for the oral or parenteral colloidal delivery systems containing BNZ for Chagas disease treatment.

## Figures and Tables

**Table 1:** The effect of the composition on the physicochemical properties assessed by DLS and rheological measurements of different LBDDS.

Samples		Diameter (nm ± SD)	PdI	ZP (mV ± SD)	*n*	*k* (Pa s)
**Free BNZ-loaded LBDDS**	A1 (SOR = 0.5)	113.9 ± 0.7	0.20	−61.8 ± 1.4	0.99	0.002
	A2 (SOR = 1.0)	87.7 ± 0.4	0.17	−60.6 ± 11.0	0.99	0.007
	A3 (SOR = 1.5)	72.3 ± 0.5	0.22	−62.7 ± 3.3	1.01	0.014
	A4 (SOR = 2.0)	ND *	ND *	ND *	0.81	0.488
	A5 (SOR = 2.5)	ND *	ND *	ND *	0.53	13.49
	A6 (OWR = 0.06)					
	A7 (OWR = 0.12)	110.2 ± 0.7	0.28	−53.6 ± 6.6	0.97	0.007
	A8 (OWR = 0.20)	87.7 ± 0.4	0.17	−37.7 ± 11.0	0.99	0.007
	A9 (OWR = 0.30)	130.8 ± 2.4	0.26	−72.4 ± 3.3	1.01	0.009
	A10 (OWR = 0.40)	74.4 ± 1.0	0.21	−89.2 ± 13.9	0.99	0.031
**BNZ-loaded LBDDS**	A1 (SOR = 0.5)	136.7 ± 0.2	0.16	−69.4 ± 6.9	1.02	0.001
	A2 (SOR = 1.0)	90.3 ± 0.4	0.16	−70.5 ± 7.0	0.91	0.009
	A3 (SOR = 1.5)	89.4 ± 1.1	0.20	−60.8 ± 4.0	0.99	0.015
	A4 (SOR = 2.0)	ND *	ND *	ND *	0.89	0.219
	A5 (SOR = 2.5)	ND *	ND *	ND *	0.65	3.106
	A6 (OWR = 0.06)					
	A7 (OWR = 0.12)	81.6 ± 0.7	0.21	−64.2 ± 4.6	1.01	0.003
	A8 (OWR = 0.20)	90.3 ± 0.4	0.16	−70.6 ± 7.0	0.91	0.009
	A9 (OWR = 0.30)	80.7 ± 0.3	0.21	−89.8 ± 1.7	1.01	0.008
	A10 (OWR = 0.40)	164.6 ± 3.6	0.24	−96.7 ± 4.0	1.03	0.009

SOR, surfactant-to-oil ratio; OWR, oil-to-water ratio; PdI, polydispersity index; ZP, zeta potential; *n*, flow index; *k,* consistency index, * ND: not determined (liquid crystals lamellar type).

## Abbreviations

LBDDS	lipid-based drug delivery systems
BNZ	benznidazole
SOR	surfactant-to-oil ratio
OWR	oil-to-water ratio
SM	surfactant mixture
SPC	soy phosphatidylcholine
SO	sodium oleate
MCT	medium chain triglyceride
NE	nanoemulsion
LC	liquid crystals
HLB	hydrophilic lipophilic balance
O/W	oil-in-water
W/O	water-in-oil
CPLM	cross-polarized light microscopy
DLS	dynamic light scattering
ZP	Zeta potential
MTT	3-[4,5-dimethylthiazol-2-yl]-2,5-diphenyltetrazolium bromide
PdI	polydispersity index
EPI	emulsification by phase inversion
